# Hypoglycemia-Induced Hemiparesis in a Diabetic Woman after Childbirth

**DOI:** 10.1155/2015/210613

**Published:** 2015-04-23

**Authors:** Vera Kukaj, Fisnik Jashari, Dren Boshnjaku, Enis Istrefi, Pranvera Ibrahimi

**Affiliations:** ^1^Clinic of Neurology, University Clinical Center of Kosovo, 10000 Pristina, Kosovo; ^2^Department of Neurology, University of Pristina, 10000 Pristina, Kosovo; ^3^Department of Public Health and Clinical Medicine, Umeå University, 901 87 Umeå, Sweden

## Abstract

A 24-year-old female with type 1 diabetes mellitus presented with hemiparesis induced by hypoglycemia. She was hospitalized because she has noticed a weakness of her right hand and leg three days after childbirth. On physical examination she had an expressive dysphasia and right side hemiparesis with facial drop. Hypoglycemia is rarely associated with hemiparesis and it is often overlooked, especially when it happens in patients at higher risk of other diseases frequently associated with hemiparesis. Although sporadical cases of hypoglycemia-induced hemiparesis were reported, the clear pathophysiology behind this is not well determined. However, any individual case is important in order to increase the awareness of hypoglycemia as an important etiology of this condition.

## 1. Introduction

Hypoglycemia could have different clinical presentations [1]. While some are synonymous with hypoglycemia and allow prompt recognition and treatment, such as confusion, coma, or autonomic complaints, the others are relatively uncommon that can be mistakenly attributed to other etiologies. Hemiparesis is a rare sign of hypoglycemia; usually ischemic or hemorrhagic strokes are the first etiologies to be considered in the elderly. But it is not the case for young adults with known history of type one diabetes mellitus. However, in special patients at higher risk, such as pregnant women or early after childbirth women with hemiparesis, other etiologies, such as deep sinus venous thrombosis or reversible ischemic syndromes (Call-Fleming syndrome), are commonly associated with hemiparesis and need to be ruled out.

Hemiparesis induced by hypoglycemia has been described as far back as 1928 [2]. Recently a predilection for right side symptoms [3] and also a correlation with transient abnormal imaging findings, frequently observed in the internal capsule or splenium of the corpus callosum [2], were shown. Indeed, the pathophysiology behind hypoglycemia-induced hemiparesis is unclear; it was suggested that cerebral vasospasm, asymmetric blood flow, and selective neuronal vulnerability could be the consequence [2]. Hemiparesis is a known presentation of hypoglycemia that can be mistakenly attributed to other causes, yet, in our knowledge, no cases of young women after childbirth, presenting with hypoglycemia-induced hemiparesis, were described in current literature.

## 2. Case

A 24-year-old female with a history of type 1 diabetes was hospitalized because she has noticed a weakness of her right hand and leg three days after childbirth. This was her first delivery and the gestational age was 39 weeks. Breast-feeding was initiated immediately. She has been treated with subcutaneous insulin, administered by the nurses. There were no data of any insulin overdosing. Four hours after symptoms were initiated she was transferred from Clinic of Gynecology and Obstetrics to our Clinic (Neurology). Recently, she had a history of preeclampsia treated with Methyldopa.

She has been diagnosed with type 1 diabetes mellitus 12 years ago and is treated with insulin therapy. On her last regular visit in November 2014 the glycated hemoglobin (HbA1c) was 7.1%.

On physical examination she had an expressive dysphasia, right side hemiparesis with facial drop, and muscle weakness on the right arm and leg (2/5) with decreased reflexes (+1). She had no ataxia or numbness. Sensibility was not affected. NIH stroke score (NIHSS) at admission was 9. Systolic blood pressure was 130 mmHg and diastolic blood pressure was 85 mmHg; heart rate was regular with frequency of 72 beats per minute. Body temperature was 37.2°C, and Glasgow Coma Scale (GCS) was 14 (E4V4M6). There were no other remarkable findings on physical examination.

Laboratory findings on admission showed hypoglycemia (glucose value was 2.1 mmol/L); liver and renal function tests were normal. Also, the level of cholesterol, triglycerides, electrolytes (calcium, sodium, potassium, and phosphate), sedimentation rate, and complete blood count (CBC) were normal, except for a microcytic anemia.

Her symptoms started to resolve quickly after 40 mL of dextrose 50% was administered intravenously. On the next morning her neurological examination was normal and the neurological deficit was no longer apparent; NIHSS was 0. The blood glucose level was 8.5 mmol/L. The endocrinologist was consulted and the doses of insulin were adjusted. Brain MRI, MRA, and MRV were performed on the next day morning in order to exclude sinus venous thrombosis or other brain diseases. No ischemic or hemorrhagic lesion was observed; also the circulation in the venous sinuses was preserved. EEG was performed to exclude abnormal electric discharge, and it showed normal brain activity.

Hemiparesis as a sign of hypoglycemia and symptoms relieve after glucose administration, accomplished Whipple's triad and supported the diagnosis of hemiparesis induced by hypoglycemia.

## 3. Discussion

We have reported a case of a 24-year-old female early after childbirth with a history of type 1 diabetes, presenting with right side hemiparesis and hypoglycemia. Since hemiparesis is a known but very rare consequence of hypoglycemia, other life-threatening conditions such as ischemic or hemorrhagic stroke and sinus venous thrombosis need to be ruled out.

Hypoglycemic brain injury is widely studied in patients with hypoglycemic coma showing involvement of cerebral cortex, basal ganglia, and hippocampus [4]. In patients presenting with hemiparesis the internal capsule and splenium were often affected, suggesting that these areas are more vulnerable to hypoglycemia. Five-hour delay in performing brain imaging may be the reason of normal results in our case. Previous studies have shown a predilection of right side symptoms in patients with hypoglycemia-induced hemiparesis [3], also confirmed in our case.

At present, the pathophysiology behind hypoglycemia-induced hemiparesis is unclear. Mechanisms about hypoglycemia-induced brain damage may involve cytotoxic edema, shrinkage of the extracellular space, and failure of ionic pumps of the cell membrane after energy depletion [5]. Disruption of the protein synthesis in the superficial layers of the cortex and basal ganglia is another hypothesis [6, 7].

Local neurological deficit may also occur after seizures. Plum et al. observed a two-to-threefold increase in glucose utilization during seizure discharges and suggested paralysis that follows (Todd postepileptic paralysis) might be a result of neuronal depletion of glucose and an increase of lactic acid [8]. In our case there were no data of seizure and the EEG showed normal brain activity.

Patients with diabetes have nearly twice the risk of depression compared to the general population. Depression has been associated with poorer adherence to self-care regimens [9] or insulin overdosing [10]. In our reported case, we have not detected any sign of depression. Furthermore, nurses did administer insulin in the last week, and there was no report of accidental insulin overuse.

Our presented case highlights the need for a closer monitoring of diabetic mothers after childbirth. Since there is a physiological need for more insulin than usual during pregnancy (up to 2-3 times more), pregnant woman needs to control the successively increased insulin need through frequent blood glucose measures and continuous insulin dose increase combined with a structured diet and activity plan [11]. Immediately after delivery the insulin need decreases, often to less than the pregestational levels [12] approximately 60% of the prepregnancy dose, owing to lack of placental hormonal influence [13]. However, there are many contributors placing the new mothers in a vulnerable situation of suffering unstable glycaemia and more hypoglycemic episodes after childbirth, including fluctuating glucose levels during breastfeeding [13], increased level of anxiety, and the downprioritizing of their own needs in favor of the child's [14].

Finally, hemiparesis is a rare but serious consequence of hypoglycemia that could result in permanent neurological damage if unrecognized. Even in other potential causes of hemiparesis such as ischemic stroke, hypoglycemia increases the susceptibility of brain tissue to hypoxia. Therefore blood glucose assessment is of crucial importance in each individual patient presenting with hemiparesis. Also, it is imperative to consider hypoglycemic hemiparesis in the differential diagnosis of stroke.

In conclusion, there is need for increasing awareness of these serious consequences in diabetic patients. Prompt measurement of plasma glucose and review of diabetes history should be performed for rapid identification of hypoglycemia as a likely cause of hemiparesis.
